# Induction of a Specific Humoral Immune Response by Nasal Delivery of Bcla2_ctd_ of *Clostridioides difficile*

**DOI:** 10.3390/ijms21041277

**Published:** 2020-02-14

**Authors:** Ana Raquel Maia, Rodrigo Reyes-Ramírez, Marjorie Pizarro-Guajardo, Anella Saggese, Pablo Castro-Córdova, Rachele Isticato, Ezio Ricca, Daniel Paredes-Sabja, Loredana Baccigalupi

**Affiliations:** 1 Dipartimento di Biologia, Università di Napoli Federico II, Via Cinthia 26, 80126 Napoli, Italy; raqueldiasmaia@outlook.com (A.R.M.); ornellasaggese2010@libero.it (A.S.); isticato@unina.it (R.I.); ericca@unina.it (E.R.); 2 Microbiota-Host Interactions and Clostridia Research Group, Departamento de Ciencias Biológicas, Facultad de Ciencias de la Vida, Universidad Andrés Bello, Avenida Republica 330, 8370186 Santiago, Chile; rodrigoreyesr1992@gmail.com (R.R.-R.); marjorie.pizarrog@gmail.com (M.P.-G.); pablo.castro.cordova@gmail.com (P.C.-C.); 3 Millennium Nucleus in the Biology of Intestinal Microbiota, Avenida Republica 330, 8370186 Santiago, Chile; 4 Dipartimento di Medicina Molecolare e Biotecnologie Mediche, Università di Napoli Federico II, Via Pansini 5, 80131 Napoli, Italy

**Keywords:** spore adsorption, spore antigens, *Bacillus subtilis*, spore surface display, mucosal vaccination

## Abstract

*Clostridioides difficile*, formerly known as *Clostridium difficile*, is a spore-forming bacterium considered as the most common cause of nosocomial infections in developed countries. The spore of *C. difficile* is involved in the transmission of the pathogen and in its first interaction with the host; therefore, a therapeutic approach able to control *C. difficile* spores would improve the clearance of the infection. The C-terminal (CTD) end of BclA2, a spore surface protein of *C. difficile* responsible of the interaction with the host intestinal cells, was selected as a putative mucosal antigen. The BclA2 fragment, BclA2_CTD_, was purified and used to nasally immunize mice both as a free protein and after adsorption to the spore of *Bacillus subtilis*, a well-established mucosal delivery vehicle. While the adsorption to spores increased the in vitro stability of BclA2_CTD_, in vivo both free and spore-adsorbed BclA2_CTD_ were able to induce a similar, specific humoral immune response in a murine model. Although in the experimental conditions utilized the immune response was not protective, the induction of specific IgG indicates that free or spore-bound BclA2_CTD_ could act as a putative mucosal antigen targeting *C. difficile* spores.

## 1. Introduction

*Clostridioides difficile* is a Gram-positive, spore-forming, and obligate anaerobe gastrointestinal bacterium, currently considered the most common cause of hospital-acquired infectious diarrhea in the developed world [1]. *C. difficile* spores are transmitted by the oro-fecal route and, once in the gut, interact with the intestinal cells of the host and persist in the spore form. In the gut, the spores can germinate and colonize the intestine when the conditions are favorable, i.e., when the number of other intestinal bacteria is reduced, for example, by an antibiotic treatment. Germination-derived vegetative cells of *C. difficile* then produce virulence factors, such as the TcdA and TcdB toxins, that induce a strong immune response and cause *C. difficile* infection (CDI) [2]. CDI produces a spectrum of clinical responses that can range from asymptomatic colonization to mild or severe diarrhea, pseudomembranous colitis, toxic megacolon, bowel perforation, sepsis, and possible death. In the last twenty years, the severity of CDI increased worldwide due to the emergence of new hyper-virulent strains such as R20291 [3]. These strains are resistant to broad-spectrum antibiotics and contain mutations in a negative regulator of the expression of the TcdA and TcdB toxins, thus increasing toxin production [4] and host mortality rate [5].

The antibiotics vancomycin and metronidazole have been the first choices of treatment for CDI for about 30 years [6]. Nowadays, in some CDI cases the patients do not respond to the antibiotics while in some other cases they undergo recurrence of the infection after a first episode [7]. Recurrence of CDI can affect up to 35% of the patients and largely increases after a second and a third episode [8]. The reduced efficacy of antibiotics has stimulated the development of new potential therapeutic options. Vaccines developed to target *C. difficile* toxins [9,10] have not been successful so far, while fecal microbiota transplantation [11,12] and probiotic therapies [13], although very promising, are still under investigation. Since *C. difficile* spores have an essential role in the transmission of the pathogen and in its first interaction with the intestinal cells, new anti-CDI treatments must focus on the spore and on its interaction with the host cells.

*C. difficile* spores are structurally similar to those of other spore formers and are characterized by a dehydrated cytoplasm (core) surrounded by protective layers, the peptidoglycan-like cortex, the proteinaceous coat and the outermost exosporium, rich in glycoproteins [14]. The BclA family of collagen-like glycoproteins are homogeneously distributed in the exosporium of *C. difficile* spores. Two members of the family, BclA2 and BclA3, have been identified as responsible of the formation of hair-like projections on the spore surface of the hypervirulent strain R20291 [15]. Both BclA2 and BclA3 are involved in the interaction with intestinal epithelial cells [15] and, therefore, are potential targets of new anti-CDI treatments. BclA2 protein of *C. difficile* R20291 is a 47.8 kDa protein, organized into three domains: (i) an N-terminal domain (NTD) anchored to the exosporium; (ii) a collagen-like domain; (iii) and a C-terminal domain (CTD) exposed to the exterior [16].

In this study, the BclA2 protein of *C. difficile* R20291 was evaluated as a potential antigen to be used to develop a mucosal vaccination strategy against CDI. The 131 amino acid CTD domain of BclA2 (BclA_CTD_) was selected as a candidate mucosal antigen. BclA2_CTD_ was displayed on the surface of *Bacillus subtilis* spores, a well-characterized mucosal vaccine delivery system [17,18], to increase its stability and favor its delivery. Free and spore-adsorbed BclA2_CTD_ were nasally administered to mice and tested for specific anti-BclA2_CTD_ immune response.

## 2. Results

### 2.1. Purified Bcla2_ctd_ Has Low Stability

**Figure 1:**
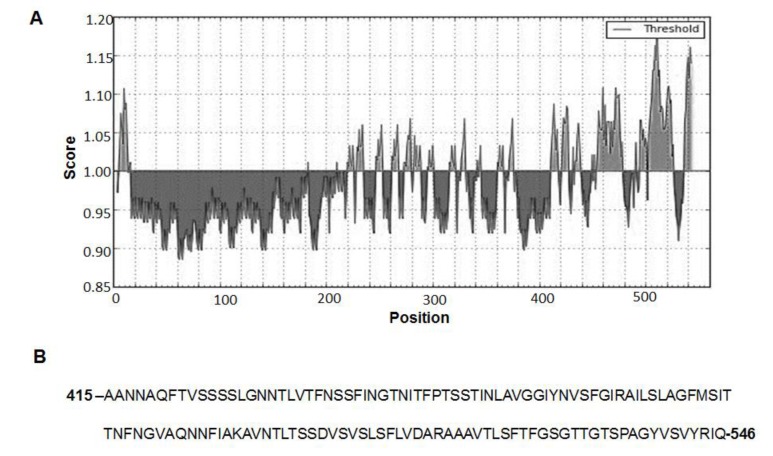
In silico analysis of BclA2 from *Clostridioides difficile* R20291. (**A**) Analysis of the B cell epitope propensity score (Kolaskar & Tongaonkar Antigenicity Method from Immune Epitope Database) of BclA2. The X- and Y-axes represent the sequence position and antigenic propensity score, respectively. The threshold value was generated by default by Immune Epitope Database (http://tools.iedb.org/bcell/). The regions above the threshold are antigenic. (**B**) Amino acid sequence of the 131 residues at the C-terminal end of BclA2 identified as a potential antigenic region and indicated as BclA2_CTD_ by Kolaskar & Tongaonkar Antigenicity Method.

**Figure 2:**
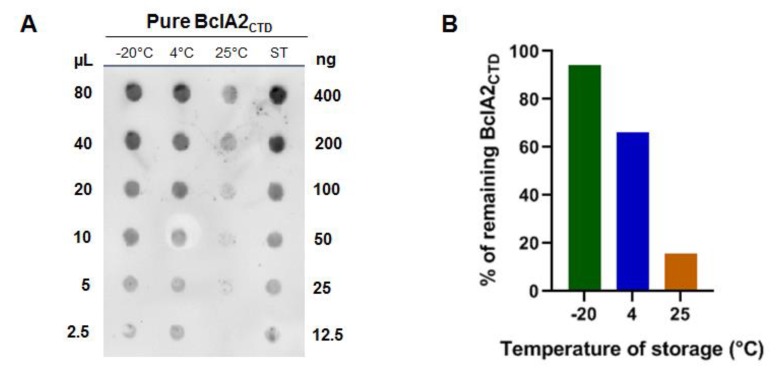
Stability of purified BclA2_CTD_. (**A**) Dot blotting of purified BclA2_CTD_ stored for 2 d at −20, +4, or at +25 °C with anti-6x His antibody. The protein stored at −80 °C right after purification (ST) was considered as standard. Loaded volumes of purified protein (μL) and known amount of the standard (ng) are indicated. (**B**) Plot of the densitometric analysis of dot blot experiment of panel A, considering the amount of protein stored at −80 °C as 100%.

First, we used an in silico approach, based on the analysis of the physicochemical properties of amino acid residues and their frequency of occurrence in experimentally known segmental epitopes in the amino acid sequence of BclA2 (Kolaskar and Tongaonkar Antigenicity Method) [19]. The 131 amino acid residues corresponding to the C-terminal end of BclA2 (BclA2_CTD_) was identified as a putative antigenic domain for the high score as B cell epitope [19] and as T cell MHC-I and MHC-II epitope [20,21] (Figure 1). Therefore, His-tagged BclA2_CTD_ was overexpressed in *Escherichia coli* BL21(DE3) and purified by affinity chromatography with Ni-sepharose columns as described in the Methods section. Next, to evaluate the stability of BclA2_CTD_, 800 ng of the purified protein suspended in phosphate buffer (PBS) pH 7.0 was incubated for 48 h either at −20, +4, or +25 °C and compared to the same amount of protein stored in the same buffer at −80 °C by dot blotting experiments with anti-His antibody (Figure 2A). Results of the densitometric analysis of the dot blot (Table S1) were plotted and showed that while storage at −20 °C caused only a minimal decrease in the amount of protein detected by the antibody, storage at 4 and 25 °C caused over 30% and 80% decrease, respectively (Figure 2B).

### 2.2. Bcla2_ctd_ Is Efficiently Displayed on B. Subtilis Spores

**Figure 3:**
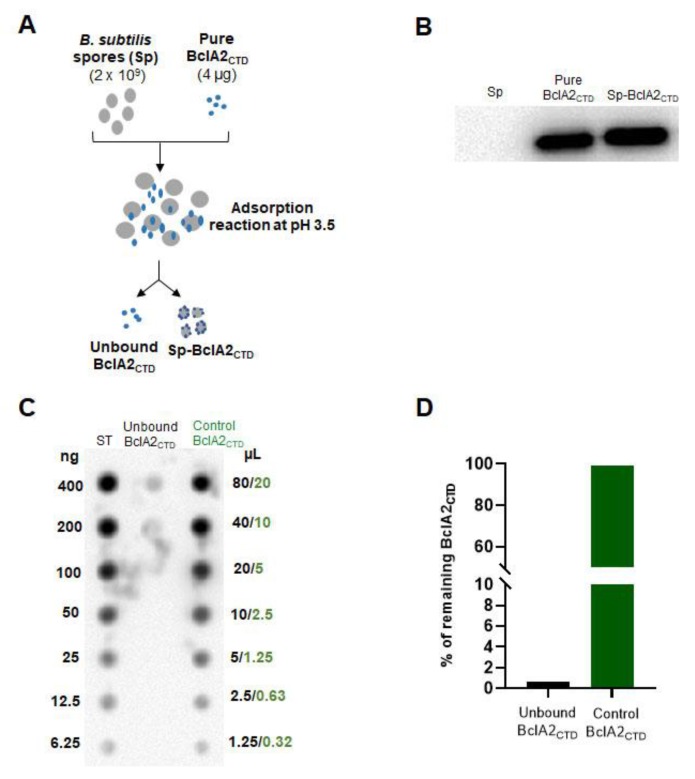
Adsorption of BclA2_CTD_ on *Bacillus subtilis* spores. (**A**) Schematic representation of the adsorption reaction. (**B**) Western blotting of spore-extracted proteins after the adsorption reaction with anti-6xHis antibody. *B. subtilis* spores (Sp) and pure BclA2_CTD_ were used as negative and positive controls, respectively. (**C**) Dot blot analysis of the supernatant of the adsorption reaction. Loaded volumes (μL) of the supernatant fraction (unbound BclA2_CTD_) and of the pure protein incubated in the same condition of adsorption (control BclA2_CTD_) are indicated in black and green, respectively. Known amounts (ng) of the pure protein incubated at −80 °C (ST) were used as standard. (**D**) Plot of the densitometric analysis of the dot blot experiment of panel C, considering the amount of protein stored at −80 °C as 100%.

In order to increase the stability of BclA2_CTD_, the antigen was adsorbed on *B. subtilis* spores, as previously reported for other antigens [22,23]. Purified BclA2_CTD_ (4 µg) was incubated with purified spores (2 × 10^9^) of the *B. subtilis* strain PY79 [24] as schematically shown in Figure 3A. The reaction mixture was then fractionated by centrifugation and pellet (containing spores and spore-bound BclA2_CTD_) and supernatant (containing unbound, free BclA2_CTD_) fractions analyzed. Spores in the pellet fraction were used to extract surface proteins that were then analyzed by Western blotting with anti-6xHis antibody. As shown in Figure 3B, BclA2_CTD_ was extracted from *B. subtilis* spores adsorbed with the purified protein, indicating that BclA2_CTD_ was adsorbed to purified spores. The supernatant fraction of the adsorption reaction was then analyzed by dot blotting with anti-6xHis antibody to measure the amount of BclA2_CTD_ left unbound and evaluate the efficiency of adsorption (Figure 3C), as previously reported [25]. In parallel, the same amount of purified BclA2_CTD_ used in the adsorption reaction (4 µg) was incubated for 1 h in PBS at pH 3.5 and analyzed by dot blotting with anti-6xHis antibody, showing no degradation under those conditions (Figure 3C). Results of the densitometric analysis of the dot blot (Table S2) were plotted and showed that 0.4% of the BclA2_CTD_ used in the adsorption reaction was left unbound, indicating that more than 99% (corresponding to 3.9 µg) of BclA2_CTD_ was adsorbed to *B. subtilis* spores (Figure 3D). These results are consistent with previously reported efficiency of adsorption for other proteins and antigens [23].

### 2.3. Bcla2_ctd_ Is Stabilized by the Adsorption on B. Subtilis Spores

**Figure 4:**
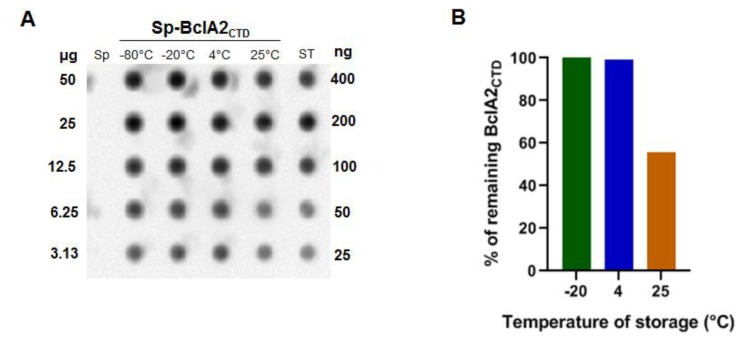
Stability of spore-adsorbed BclA2_CTD_. (**A**) Dot blot of the protein extracted from spores adsorbed with BclA2_CTD_ stored for 2 d at −80, −20, +4, or +25 °C, using an anti-6xHis antibody. Proteins extracted from *B. subtilis* spores (sp) were used as negative control. Known amounts of the pure protein stored at −80 °C immediately after its purification (ST) were used as controls (right). (**B**) Plot of the densitometry analysis of dot blot experiment of panel A, considering the amount of protein extracted from spores stored at −80 °C as 100%.

To evaluate the stability of spore-adsorbed BclA2_CTD_, 2 × 10^9^ spores displaying 4 µg of BclA2_CTD_ were centrifuged and incubated as pellets for 48 h either at −20, +4, or +25 °C. Pellets incubated at −80 °C were used as control. The spore surface proteins were then extracted and analysed by dot blot experiments with anti-6xHis antibody, and the amount of BclA2_CTD_ was compared to known amounts of pure protein stored at −80 °C immediately after its purification as standard (ST) (Figure 4A). Results of the densitometric analysis of the dot blot (Table S3) were plotted and showed that the amount of BclA2_CTD_ recognized by the antibody was not decreased after incubation at −20 or +4 °C; however, a 50% decrease in BclA2_CTD_ recognition was observed after incubation at +25 °C (Figure 4B).

The comparison of the results of Figure 2 and Figure 4 clearly indicate that upon adsorption to spores the heat stability of BclA2_CTD_ was increased, suggesting that the binding to the spore protects the antigen from degradation.

### 2.4. Adsorption of Bcla2_ctd_ Increases Spore Adherence to Caco-2 Cells

**Figure 5:**
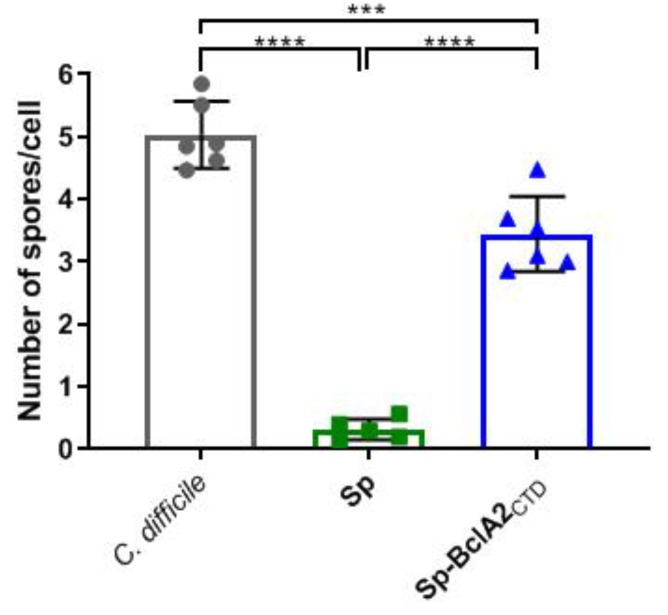
Adherence of spores to human intestinal cells. Caco-2 cells were differentiated for 8 d and infected with spores of *C. difficile* R20291, *B. subtilis* (Sp), and *B. subtilis* spores adsorbed with BclA2_CTD_ (Sp-BclA2_CTD_). The number of adhered spores and cells was counted in 10 microscopic fields on phase-contrast microscopy. The data represent the mean of three independent experiments, and the error bars are the standard error of the mean. The groups were compared with one-way ANOVA Turkey’s multiple comparison test, and statistical differences (*p* < 0.05) are indicated by asterisks.

The adherence of *B. subtilis* spores adsorbed with BclA2_CTD_ to differentiated Caco-2 cells was evaluated and compared to the adherence pattern of *C. difficile* R20291 and *B. subtilis* spores. While the adherence of R20291 spores reached 5 spores/cell, *B. subtilis* spores had an average adherence of less than 1 spore/cell (Figure 5). The adherence of *B. subtilis* spores to Caco-2 cells was significantly (*p* < 0.0001) increased upon adsorption of BclA2_CTD_ (Figure 5), suggesting that BclA2_CTD_ contributes to adherence of the spores to intestinal epithelial cells.

### 2.5. Intranasal Immunization

**Figure 6:**
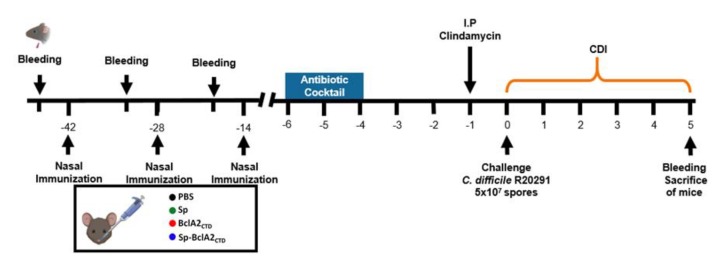
Overview of the experimental design schematics for the prevention of *C. difficile* infection in a murine model. C57BL/6 mice were nasally immunized 3 times (42, 28, and 14 d before challenge with *C. difficile* R20291 spores) with PBS, spores of *B. subtilis* PY79 (Sp), pure BclA2_CTD_, or spores of *B. subtilis* adsorbed with BclA2_CTD_ (Sp-BclA2_CTD_). Prior to the infection, the animals were submitted to an antibiotic cocktail (4 to 6 d before challenge) and clindamycin administration (1 d before challenge). On day 0 mice were infected with 5 x 10^7^ spores of *C. difficile* R20291 and were monitored for *C. difficile* infection (CDI) symptoms from day 0 to day 5. Serum was collected one day before each immunization as well as on the day of sacrifice (days 0, 13, 27, and 47).

**Figure 7:**
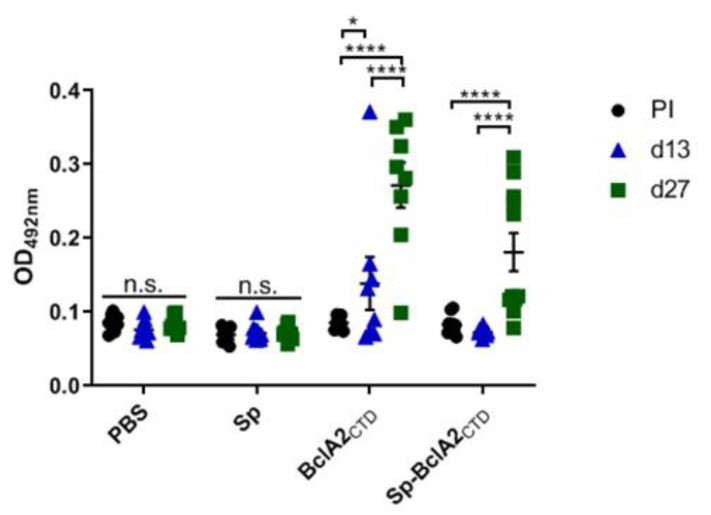
Immunogenicity of BclA2_CTD_ protein and Sp-BclA2_CTD_ in mice after nasal immunizations. IgG anti-BclA2_CTD_ levels were measured by ELISA in the serum of mice on days 0 (pre-immune serum, PI), 13 (d13), and 27 (d27) after the beginning of the experiment (one day before each immunization). Results are reported as optical density (OD) units at 492nm. The geometric mean (± standard error of the mean) for each group is shown. IgG titers of each group were compared between d0 (PI), d13, and d27 with one-way ANOVA Turkey’s multiple comparison tests, and statistical differences (*p* < 0.05) are indicated by asterisks.

*B. subtilis* spores presenting BclA2_CTD_ on their surface, and the pure BclA2_CTD_ peptide, were used for mucosal immunization experiments in mice. In particular, the animals were assigned into four experimental groups (*n* = 11), according to the immunization regimen: (i) PBS; (ii) 2 × 10^9^ *B. subtilis* PY79 spores (Sp); (iii) 2 × 10^9^ *B. subtilis* spores adsorbed with BclA2_CTD_ (Sp-BclA2_CTD_); and (iv) 4 μg of purified BclA2_CTD_. The animals were nasally immunized 42, 28, and 14 d before the challenge with *C. difficile* R20291. Animal serum was collected one day before each immunization and on the time of sacrifice (Figure 6). The production of specific anti-BclA2_CTD_ IgG was assessed by ELISA (Figure 7).

Animals treated with purified BclA2_CTD_ showed a significant increase of IgG titers mainly after the second immunization (*p* < 0.0001). The same result was observed for animals immunized with Sp-BclA2_CTD_ (*p* < 0.0001), suggesting that the spore-bound antigen is correctly assembled and able to induce a response. However, no differences were observed between the purified and the spore-bound antigen, indicating that *Bacillus* spores did not have an adjuvant effect.

**Figure 8:**
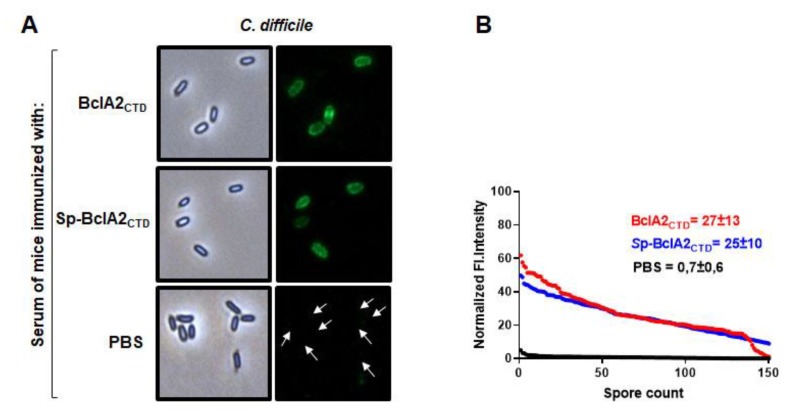
Recognition of *C. difficile* spores by the serum of animals immunized with purified BclA2_CTD_ and Sp-BclA2_CTD_. (**A**) Spores of *C. difficile* R20291 were incubated 1 h with serum (1:100) of mice immunized with BclA2_CTD_ and Sp-BclA2_CTD_ or PBS, as indicated. (**B**) The immunofluorescence micrographs are depicted in fluorescence intensity (FI.Intensity) profiles provided by fluorescence microscopy images using ImageJ. The values shown in the graphs are the average ± standard error of the fluorescence intensity from 150 spores. The secondary antibody is anti-mouse conjugated with Alexa 488.

To explore the pattern of recognition of *C. difficile* spores by the raised antibodies, spores of *C. difficile* R20291 were incubated with the serum of mice immunized with purified BclA2_CTD_, Sp-BclA2_CTD_, or PBS and stained with secondary anti-mouse IgG Alexa 488 conjugate and then analysed by fluorescence microscopy (Figure 8A). No immunofluorescence reactivity was observed in serum of mice immunized with PBS (control). By contrast, significant immunofluorescence was evidenced by antibodies produced by immunizing mice with purified BclA2_CTD_ and spore-adsorbed BclA2_CTD_ (average fluorescence intensity was 88- and 89-fold higher than the control, respectively) (Figure 8B). Altogether, these results indicate that nasal immunization with either purified free BclA2_CTD_ or spore-bound BclA2_CTD_ was capable of inducing BclA2_CTD_-specific antibody production.

### 2.6. Effect of Nasal- Bcla2_ctd_ Immunization Against C. Difficile R20291 Infection

**Figure 9:**
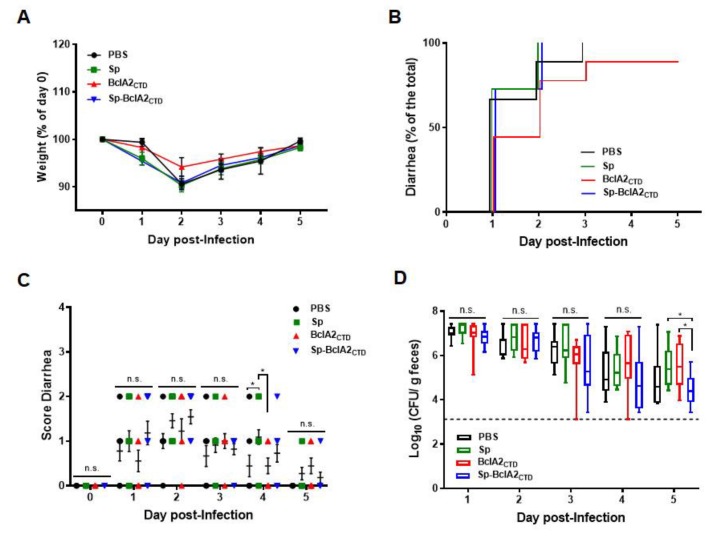
Challenge experiments. C57BL/6 mice were nasally immunized with PBS, spores of *B. subtilis* (Sp), Sp-BclA2_CTD_, and purified BclA2_CTD_ (9 to 11 animals each group) and challenged with *C. difficile* R20291 spores. Mice were monitored in the following 5 d after infection for (**A**) weight loss, presented as the relative % of the weight to the day of infection (day 0); (**B**) time of occurrence of diarrhea, presented as the relative % of diarrhea in a group to the total mice; (**C**) score of diarrhea per day; and (**D**) number of *C. difficile* spores in feces (represented as log_10_ CFU/g of feces). Differences between groups were assessed by two-way ANOVA Turkey’s multiple comparison tests, and statistical differences (*p* < 0.05) are indicated by asterisks. The bars are the geometric mean ± standard error of the mean.

Next, we assessed whether nasal immunization of mice protected against a *C. difficile* challenge. Weight loss and presence of diarrhea after infection, characterized by high spore-load in feces and colonic tissues, are expected symptoms of CDI. We measured weight loss progression over the five days after challenge and assigned a score of diarrhea according to its severity. Results demonstrate that nasal immunizations did not halt the weight loss in the subsequent 5 d after infection (Figure 9A). However, despite not being statistically significant, the animals immunized with purified BclA2_CTD_ showed a delayed onset of diarrhea, contrary to the other groups in which more than 50% of the animals already had diarrhea at day 1 after the infection (Figure 9B). Moreover, when comparing the score of diarrhea between groups (Figure 9C), it is possible to observe that on day 1 after infection, the group of animals immunized with purified BclA2_CTD_ had a reduced score of diarrhea even though not statistically significant. Interestingly, on day 3 after infection, the animals immunized with adsorbed spores showed a reduction in *C. difficile* spore load in feces (Figure 9D), which became significant on day 5 after infection (*p* = 0.0215 and *p* = 0.0306 when compared with Sp and purified free BclA2_CTD_).

**Figure 10:**
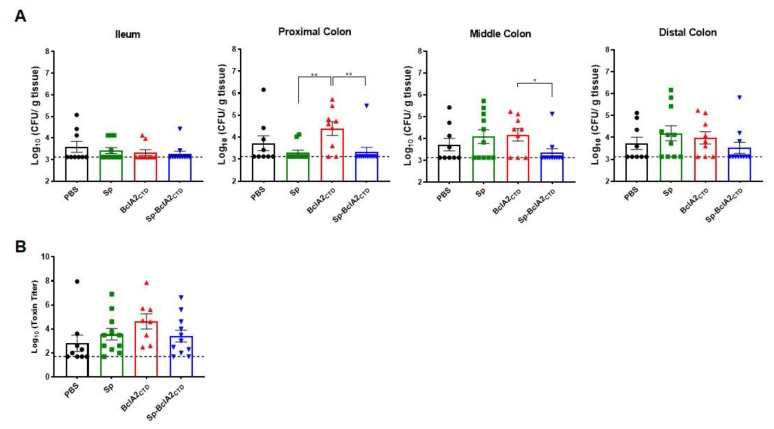
Challenge experiments on colonic tissues. (**A**) The loads of *C. difficile* spores in the ileum, proximal colon, middle colon and distal colon were evaluated upon sacrifice as log_10_ CFU/g of tissue. (**B**) The cecum content toxicity was measured and represented as log_10_ toxin titer. The differences between groups are evaluated with Mann–Whitney tests between groups for each tissue and one-way ANOVA Turkey’s multiple comparison tests for toxin titers. Statistical differences (*p* < 0.05) are indicated by asterisks The bars are the geometric mean mean ± standard error of the mean.

No significant differences between groups in *C. difficile* spore-load in the ileum or distal colon were observed (Figure 10A). However, animals immunized with Sp-BclA2_CTD_ showed a slight decrease in spore-load in the proximal and middle colon in comparison with the other groups. This reduction became significant in comparison with the animals immunized with purified BclA2_CTD_ (*p* = 0.0041 for proximal colon and *p* = 0.0324 for middle colon). However, there were no significant differences between groups on the cytotoxic titers of the cecal content (Figure 10B), meaning that the immunizations did not halt spore colonization inside the cecum.

## 3. Discussion

In recent years, the emergence of antibiotic-resistant, hypervirulent strains has urged the scientific community to develop new therapeutic strategies to fight *C. difficile* infection (CDI) [26,27]. New treatments based on probiotics or fecal transplants, although very promising, are still at an experimental stage, while vaccines targeting *C. difficile* cells [28] or toxins [29] have been so far unsuccessful. Since CDI is transmitted by spores that are also responsible of the initial interaction with the host, alternative vaccination strategies have to focus on the spore and on the surface proteins involved in the interaction with the intestinal epithelial cells. Most *C. difficile* genomes have a pseudogenized version of *bclA1*, leading to the expression of a 48 aa polypeptide sequence of the amino-terminal domain that is localized to the exosporium layer of *C. difficile* spores [16]. In this context, recent work has shown that intraperitoneal immunization with the spore surface protein BclA1 induced a strong, specific immune response but failed to provide protective immunity [30]. Two additional collagen-like exosporium proteins are also encoded in *C. difficile* genomes, BclA2 and BclA3, which to date have not been evaluated as vaccine candidates. In this study, a fragment of the spore surface protein BclA2 (BclA2_CTD_) was identified as a potential antigen and tested as a mucosal vaccine.

The mucosal surfaces of the host are the most common route of entry used by pathogens, including *C. difficile*; therefore, it is important for a vaccine to elicit an immune response at the mucosal surfaces. However, only few mucosal vaccines are currently licensed, mostly because of the low immunogenicity of mucosal antigens and to antigen degradation during storage or at the mucosal surfaces [31]. One strategy that we used to avoid or reduce antigen degradation was to display BclA2_CTD_ on *B. subtilis* spores, a mucosal vaccine delivery system previously tested with other antigens [22,23]. Our data demonstrate that BclA2_CTD_ was very efficiently displayed in *B. subtilis* spores, and the interaction with the spore surface layers reduced BclA2_CTD_ degradation during storage, as previously reported for other antigens and enzymes [32]. However, in spite of the low in vitro stability of the free BclA2_CTD_ with respect to the spore-bound antigen, both forms of BclA2_CTD_ induced similarly strong humoral immune responses when administered to mice by the nasal route.

Upon immunizations, mice were subjected to a challenge with the epidemically relevant *C. difficile* strain R20291. Although no obvious protective effects were observed on mice, the *C. difficile* spore load in feces showed a tendency to decrease three days after infection in animals immunized with the spores displaying BclA2_CTD_. In addition, these same groups of animals also showed a reduction of spore load in the proximal and middle colon, possibly a consequence of *C. difficile* spore opsonization by anti-BclA2_CTD_ antibodies. A similar lack of protection efficacy was previously observed upon immunization with the BclA1 and SleC, but not with the cysteine-rich proteins CdeC and CdeM [30], suggesting that the collagen-like proteins BclA1 and SleC are not good vaccine candidates. A possible explanation of the lack of clear signs of protection observed in our results is that the dose of antigen might not have been high enough to reduce CDI symptoms and *C. difficile* spore germination inside the mice cecum. Indeed, works using *B. subtilis* spores adsorbed with heterologous antigens that induced a protective effect in nasally immunized mice applied a higher dose regimen and frequency than we did in this work. Plus, some works have also used the same immunization regimen and induced both humoral and cellular immune responses. However, they aimed to have a stronger effect in the nasal mucosa and not in the gut, since they used *Mycobacterium tuberculosis* antigens or H5N1 virions [33,34].

Additional in vivo experiments with higher doses of antigen and/or using the oral route of immunization would be needed to address this point. Even though no protection against a challenge was observed, the identification of an efficient antigen for *C. difficile* spores, active when administered by the mucosal route, is a promising result that could open to a new vaccination strategy against CDI.

## 4. Materials and Methods

### 4.1. Bacterial Strains and Spore Purification

*B. subtilis* PY79 [24] was used for the adsorption reaction. The hypervirulent *C. difficile* strain R20291 was used for the challenge experiment. *E. coli* strain BL21 (DE3) (ThermoFisher Scientific, Waltham MA, USA) was used for BclA2_CTD_ overexpression.

Sporulation of *B. subtilis* was induced by the exhaustion method [35]. Briefly, after 35 h of growth in Difco Sporulation (DS) medium at 37 °C with vigorous shaking, spores were collected, washed, and purified. The purification was performed using KCl 1M, lysozyme 10mM, NaCl 1M, SDS 0.05%, and several washes with water.

*C. difficile* spores were purified as described elsewhere [36]. Briefly, 1:100 dilution of *C. difficile* overnight culture was plated onto a 70:30 medium (63 g Bacto peptone (BD Difco), 3.5 g proteose peptone (BD Difco), 0.7 g ammonium sulfate, 1.06 g Tris Base, 11.1 g brain heart infusion extract (BD Difco), and 1.5 g yeast extract (BD Difco) for 1 L) and incubated under anaerobic conditions for 7 d at 37 °C [37]. Upon incubation, the surface of the plates was scraped up with ice-cold sterile water, and the spores were gently washed five times with ice-cold sterile water in the micro centrifuge at 14,000 rpm for 5 min. After, the spores were loaded onto a 50% Nycodenz solution and centrifuged once again in the same conditions previously described. The pellet was then washed five times with ice-cold sterile water to remove Nycodenz remnants. The spores were quantified in the Neubauer chamber, and the volume was adjusted at 5 × 10^9^ spores per mL.

### 4.2. BclA2_CTD_ over-Production and Purification

The coding sequence of the BclA2 C-terminal domain (CTD) was amplified using the chromosomal DNA of *C. difficile* R20291 (396bp) as a template and oligonucleotides BclA2_CTD_sense (ggtaccccatggggatccGCAGCAAACAATGCACAATTTACAG, in lower case are the recognition sites for KpnI, NcoI, and BamHI restriction enzymes) and BclA2_CTD_anti (tctagactgcagCTATTGTATTCTATAAACTGATACATAC, in lower case are the recognition sites for XbaI and PstI restriction enzymes) to prime the reaction. Amplified DNA was cloned in pGEMT-easy (Promega, Madison, Wisconsin, USA). Subsequently, the gene of interest was cleaved with BamHI/PstI restriction enzymes and inserted in-frame to the sequence coding for an N-terminal polyhistidine tag in the expression vector pRSETA (Invitrogen) previously digested with the same enzymes. Expression of the recombinant gene was controlled by a T7 promoter inducible by lactose. The recombinant plasmid was used to transform competent cells of the *E. coli* strain BL21(DE3). The recombinant strain was grown for 16 h at 37 °C in auto-induction medium [38] to express the heterologous protein. The His-tagged BclA2_CTD_ protein was purified under native conditions using the His-Trap column (eluted with 500mM of imidazole) as recommended by the manufacturer (GE Healthcare Life Science, Merk, Darmstardt, Germany). The purified protein was desalted and concentrated with the Centricon cut-off 10kDa (Merck, Millipore, Darmstardt, Germany). The purity of the protein was verified by SDS-page and Western blot using anti-His antibodies.

### 4.3. Adsorption Reaction, Stability, and Production of Spores for Animal Immunization

Purified BclA2_CTD_ (4 µg) was added to a suspension of 2 × 10^9^ *B. subtilis* spores in 0.15 M PBS pH 3.5 at 25 °C in a final volume of 200 µL. After 1 h of incubation, free unbound BclA2_CTD_ and spore-adsorbed BclA2_CTD_ (Sp-BclA2_CTD_) were separated by centrifugation and analysed by dot and Western blotting. The stability of free and spore-adsorbed BclA2_CTD_ was assessed by exposing purified BclA2_CTD_ (suspended in phosphate buffer pH 7) and the BclA2_CTD_-adsorbed spores at different temperatures before analysis.

For mice immunization, 144 µg of purified BclA2_CTD_ was incubated with a suspension of 7.4 × 10^10^ *B. subtilis* spores in 0.15 M PBS pH 3.5 at 25 °C in a final volume of 8 mL. A total of 2 × 10^9^ spores were used for Western and dot blot analyses, and the remaining spores were aliquoted and stored at −80 °C for mice immunization.

### 4.4. Western and Dot-Blot Analyses

A total of 2 × 10^9^ *B. subtilis* spores or pure BclA2_CTD_ were suspended in extraction buffer 2x [25] incubated at 100 °C for 7 min. Proteins were loaded on SDS-PAGE gel (15%), electro-transferred to nitrocellulose filters (Amersham Pharmacia Biotech, Milano, Italy), and used for Western blot analysis. For the quantitative evaluation of the amount of BclA2_CTD_, serial dilutions of purified BclA2_CTD_, of the supernatant of the adsorption reaction or of protein extracted from spores displaying BclA2_CTD_, were analysed by dot blot experiments. Protein extraction from the spore coat was obtained by incubating 2 × 10^9^ Sp-BclA2_CTD_ in SDS 10%, DTT 1 M, and Tris-HCl pH 6.8 1.5 M 1 h at 65 °C (200 μL final volume), 2 min at 4 °C, and posterior centrifugation. Protein quantification on the dots was obtained by exposing the filters to ECL-prime (Amersham Pharmacia Biotech), and then, using Quantity One 1-D Analysis Software (Bio-Rad, Segrate, Milano, Italy), the dots were subjected to densitometric analysis. Anti-His antibodies were used both in Western and dot blot assays.

### 4.5. Adherence to Caco-2 Cells

An existing stock of Caco-2 cells in the Microbiota–Host Interaction and Clostridia Research Group at the Universidad Andrés Bello was routinely grown at 37 °C with 5% CO_2_ in Dulbecco’s modified Eagle’s minimal essential medium (DMEM) (HyClone, GE Healthcare Life Science, Merk, Darmstardt, Germany), supplemented with 10% (vol/vol) fetal bovine serum (FBS) (HyClone), penicillin (100 U/mL), and streptomycin (100 µg/mL). Spore adherence of *C. difficile* R20291, *B. subtilis* PY79, and *B. subtilis* adsorbed with BclA2_CTD_ to Caco-2 cell line was measured as previously described [39]. Briefly, Caco-2 cells were seeded onto glass coverslips in 24-well plates (4 × 10^5^ cells per well), and to obtain differentiated Caco-2 cells, cells were cultured for 8 d post-confluence, changing the medium every other day, using previously described methods [40]. Monolayers were infected with 2.5 × 10^7^ spores in 200 μL of culture medium without FBS. Cells infected with spores were incubated under aerobic conditions 3 h at 37 °C. After incubation, the unbound spores were removed by washing three times with DPBS. In order to count the number of cells, the nuclei were stained with Hoechst stain (Sigma-Aldrich, St. Louis, MO, USA) 1:1000 in PBS 10 min. After several washes with PBS and H_2_O, the coverslips were dried 15 min at 37 °C, mounted using Dako Fluorescence Mounting medium (Dako, North America, Carpinteria, CA, USA), and sealed with nail polish. Samples were analysed with an Olympus BX53 microscope. The number of spores and cells were counted, and the adherence was represented as number of spores/cell. The experiment was conducted in triplicate.

### 4.6. Animals

Pathogen-free male or female C57BL/6 mice (age 8–12 w) were obtained from a breeding colony at Facultad de Ciencias Biologicas Universidad Andres Bello (Santiago, Chile), established with animals purchased from Jackson Laboratories. Mice handling and experimental protocols were performed according to Animal ethic committee of the Faculty of Life Sciences of the Universidad Andrés Bello (Protocol number 0035/2018, project identification code: Fondef ID18/10230; approval date: 22nd january 2018; approval act code 0035/2018). 

Mice were exposed to a 12-h cycle of light and darkness, and water, bedding, and cages were previously autoclaved.

### 4.7. Immunization Regimen in Mice

Mice were randomly assigned to four experimental groups (11 animals in each group) according to the type of immunization received. The mice were intranasally immunized on days 0, 14, and 28 after the beginning of the experiment with 20 μL (10 µL per nostril) of PBS pH 7, 2 × 10^9^ spores of *B. subtilis* PY79, 2 × 10^9^ *B. subtilis* PY79 spores adsorbed with BclA2_CTD_ (Sp- BclA2_CTD_), or 4μg of pure BclA2_CTD_. The day before each immunization and on the day of the sacrifice (on 47th day), the blood was collected.

### 4.8. Animal Infection Model

Six days before infection with *C. difficile* spores, mice were exposed to an antibiotic cocktail with kanamycin (40 mg/kg body weight; Sigma-Aldrich, USA.), gentamicin (3.5 mg/kg body weight; Sigma-Aldrich, USA.), colistin (4.2 mg/kg body weight; Sigma-Aldrich, USA.), metronidazole (21.5 mg/kg body weight; Sigma-Aldrich, USA.), and vancomycin (40 mg/kg body weight; Sigma-Aldrich, USA.) for 3 d by oral administration. The antibiotic treatment was followed by intraperitoneal administration of a single dose of clindamycin (10 mg/kg) 1 d before *C. difficile* infection [41]. All animals were infected oro-gastrically with 100 μL of PBS containing 5 × 10^7^ spores of *C. difficile* strain R20291. From this point, all procedures were made inside a biosafe cabinet to contain spore-mediated transmission. Each mouse was individually housed in strerile cages, and mice had ad libitum access to food and water.

Mice conditions were daily monitored according to a scoring system. Presence and morphology of diarrhea was classified as follows: (i) normal stool (score = 0); (ii) colour/consistency change (score = 1); (iii) presence of wet tail or mucosa (score = 2); (iv) liquid stools (score = 3). A score higher than 1 was considered as diarrhea [42]. Other clinical symptoms as variations of weight, physical aspect (i.e., abnormal/hunched gait, piloerection), spontaneous behaviour (i.e., lethargy, inactivity or lack of mobility), and emaciation were monitored as described [43]. Moribund mice or mice displaying overt signs of disease were sacrificed. At the time of euthanasia, ileum, proximal, median, and distal colon were collected as well as the cecum content.

### 4.9. Quantification of Spores from Feces and Colon Samples

Fecal samples were collected daily and stored at −20 °C until spore quantification. Ten microliters of PBS was added for each mg of stool, mixed, and incubated for 30 min at room temperature. Then, 50 µL of absolute ethanol (Sigma-Aldrich) was added to 50 µL of feces and incubated for 30 min at room temperature. Samples were serially diluted and plated onto selective medium supplemented with taurocholate (0.1% *w*/*v*), cefoxitin (16 μg/mL), and l-cycloserine (250 μg/mL) (TCCFA plates). The plates were incubated anaerobically at 37 °C for 48 h, the *C. difficile* colonies were counted, and the results were expressed as the log10 (CFU/g of faeces). Proximal, median, and distal colon were collected from mice upon sacrifice and washed with PBS with a syringe. Then, they were resuspended and homogenized with 2.5 µL of PBS for each mg of tissue. Upon incubation at room temperature with absolute ethanol and serially diluted, they were plated onto TCCFA plates. The plates were incubated anaerobically at 37 °C for 48 h. Finally, the colony count was expressed as the log10 (CFU/g of tissue).

### 4.10. Evaluation of Bcla2_ctd_-Specific Igg Levels in Mice Serum

The blood collected the day before each immunization and at the time of sacrifice was incubated at 37 °C for 30 min and posteriorly centrifuged at 5000 rpm for 20 min at 4 °C. The supernatant containing the serum fraction was stored at −20 °C until use. To assess the production of IgG against BclA2_CTD_, an enzyme-linked immunosorbent assay (ELISA) was performed. Pure BclA2_CTD_ was coated onto 96-well plates at 100ng/well overnight at 4 °C. Plates were blocked with PBS–0.05% Tween-20 (PBS-T) containing 2% BSA for 1 h at 37°C. After several washes, the wells were next incubated with 1:100 of animal serum (in 1% BSA in PBS-T). The plates were incubated 2 h at 37 °C. After the removal of non-adherent IgG by several washes, the plates were incubated with secondary antibody anti-mouse HRP for 1 h at 37 °C. Finally, the colorimetric reaction was initiated upon the addition of 50 µL of reaction buffer containing 0.05 M citric acid, 0.1 M disodiumhydrogen phosphate, 2 mg/mL of *o*-phenlyendiamine (Sigma-Aldrich, USA.), and 0.015% of H_2_O_2_ (Merck, Germany). The reaction was stopped after 20 min with 25 µL of 4.5 N of H_2_SO_4_, and absorbance was measured at 492 nm. The experiment was performed in duplicate.

### 4.11. Immunofluorescence Analysis

Spores of *C. difficile* R20291 were fixed for 20 min with 3% paraformaldehyde (pH 7.4) on cover glass slides previously coated with poly-l-lysine. Coverslips were washed three times with PBS, blocked 1 h with 1% BSA, and further incubated at room temperature 1 h with 1:100 of serum from an immunized mouse with PBS, Sp-BclA2_CTD_, or purified BclA2_CTD_ (in PBS BSA 1%). The coverslips were rinsed three times with PBS and incubated 1 h at room temperature with 1:400 anti-mouse conjugated with Alexa 488 (ThermoFisher Scientific, Invitrogen, Waltham MA, USA) in PBS BSA 1% and washed three times with PBS and one time with sterile water. Finally, after drying 30 min at room temperature, the coverslips were mounted with Dako Fluorescence Mounting medium (Dako, North America), sealed with nail polish, and then analysed with Olympus BX53 fluorescence microscope. The fluorescence images were obtained with 30 ms of exposition. ImageJ software was used to quantify the fluorescence signal, as previously reported [25].

### 4.12. Cytotoxicity Assay

The toxicity of the cecum content was determined using Vero cells [44]. Briefly, 96-well flat-bottom microtiter plates were seeded with 10^5^ Vero cells/well. Mice cecum contents were weighted and then mixed with PBS (10 μL of PBS per mg of cecum content) and centrifuged (14,000 rpm 5 min). The supernatant, previously sterilized with a filter, was serially diluted in DMEM supplemented with FBS 10% and penicillum streptomycin 1%, and then 100 μL of each dilution was added to wells containing Vero cells. The plates were incubated 16 h at 37 °C, and then cell rounding was analysed. For each series of dilutions for one cecum content, the highest dilution that produced at least 80% of cell rounding under X200 magnification was considered.

### 4.13. Statistical Analysis

Prism 8 (GraphPad Software, Inc., San Diego, CA, USA) was used for statistical analysis. Normality was assessed by Shapiro–Wilk tests. For populations that did not follow a normal distribution, significance between groups was assessed by Mann–Whitney unpaired *t*-tests. Comparative analysis between groups was performed by analysis of variance with Turkey’s multiple comparison test for populations that followed a normal distribution. A *p*-value of ≤0.05 was accepted as the level of statistical significance.

## 5. Conclusions

Final goal of this work is to develop a new vaccination strategy against *Clostridium difficile* spores, the cellular form of the pathogen involved in the transmission of the pathogen and in its first interaction with the host. To this aim, we identified the C-terminal domain of the exosporium protein BclA2 (BclA2_CTD_) of *C. difficile* as an effective mucosal antigen. To increase antigen stability and facilitate its delivery, BclA2_CTD_ was efficiently exposed on *B. subtilis* spores and observed that the interaction with the spore protected the antigen from degradation during the storage. Both free and spore-adsorbed BclA2_CTD_ were used to nasally immunize mice and were both able to induce a specific humoral immune response. 

## Supplementary Materials

Supplementary materials can be found at https://www.mdpi.com/1422-0067/21/4/1277/s1.

## Abbreviations

CDI	*Clostridioides difficile* infection
BclA	*Bacillus* collagen-like protein of anthracis
NTD	N-terminal domain
CTD	C-terminal domain
BclA2_CTD_	C-terminal domain of BclA2
ST	Standard
PBS	Phosphate-buffered saline
Sp	Spores of *B. subtilis*
Sp-BclA2_CTD_	Spores of *B. subtilis* adsorbed with BclA2_CTD_
ANOVA	Analysis of variance
ELISA	Enzyme-linked immunosorbent assay
IgG	Immunoglobulin G
I.P	Intraperitoneal
Wt	Wild-type
PI	Pre-Immune
d13	Day 13
d27	Day 27
OD	Optical density
Fl.I	Fluorescence intensity
CFU	Colony forming unit
