# Supplementary figures and images for: Induction of a Specific Humoral Immune Response by Nasal Delivery of Bcla2ctd of Clostridioides difficile
Source: Int J Mol Sci. 2020 Feb 14;21(4):1277. doi: 10.3390/ijms21041277 (PMC7072882; doi:10.3390/ijms21041277)

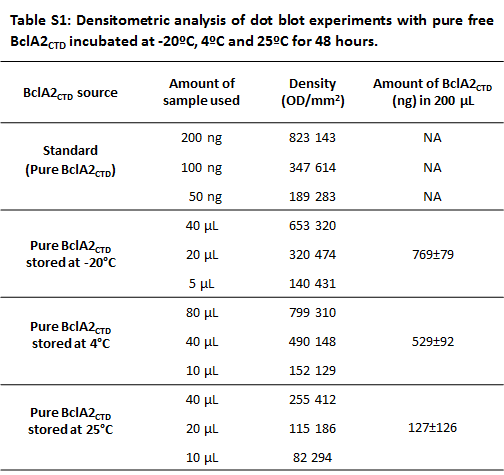

Supplement: Supplementary file 1 [file ijms-21-01277-s001.zip › TableS1.tif]

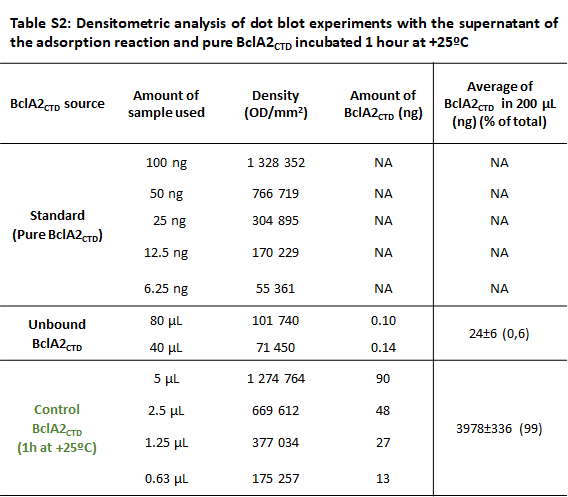

Supplement: Supplementary file 1 [file ijms-21-01277-s001.zip › TableS2.tif]

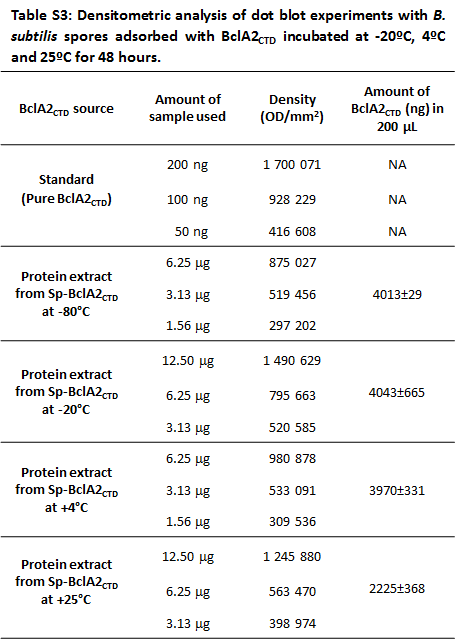

Supplement: Supplementary file 1 [file ijms-21-01277-s001.zip › TableS3.tif]
